# Complications after Gamma Knife Radiosurgery for Brain AVMs: Predictive factors for symptomatic radionecrosis

**DOI:** 10.1007/s00701-025-06532-5

**Published:** 2025-04-23

**Authors:** Popadic Branko, Amedeo Cervo, Antonio Macera, Guglielmo Pero, Giada Valente, Florian Scheichel, Camillo Sherif, Marco Picano, Marco Cenzato, Alessandro La Camera, Mariangela Piano

**Affiliations:** 1 Neuroradiology Unit, ASST Grande Ospedale Metropolitano Niguarda, Milan, Italy; 2 Karl Landsteiner University of Health Sciences, Krems, Austria; 3 Department of Neurosurgery, University Hospital St. Poelten, St. Poelten, Austria; 4 Department of Oncological, Radiological and Anatomopathological Sciences, Sapienza University of Rome, Rome, Italy; 5 Department of Neurosurgery, ASST Grande Ospedale Metropolitano Niguarda, Milan, Italy

**Keywords:** AVM, Gamma Knife, Endovascular treatment, Complication

## Abstract

**Purpose:**

The aim of this study is to investigate complications after Gamma Knife Radiosurgery (GKRS) for AVMs and predictive factors for symptomatic radionecrosis.

**Methods:**

A retrospective single centre study on AVMs treated with GKRS between 2008 and 2016 was performed.

**Results:**

A total of 209 patients were included. AVM obliteration was seen in 70%, while radiation induced changes (RIC) were detected in 45%. Symptomatic radionecrosis was found in 13 patients (6.2%). Furthermore, 12 patients (5.7%) experienced latent period haemorrhage. Predictors of symptomatic radionecrosis were 12 Gy volume (*p* = 0.007), RIC grade (*p* =  < 0.0001) and ≥ 2 endovascular treatments (*p* = 0.001) in univariate analysis, while age (*p* = 0.043), RIC grade (*p* = 0.0002) and ≥ 2 endovascular procedures (*p* = 0.002) were identified in multivariate analysis.

**Conclusion:**

Complication after GKRS for AVMs were latent period haemorrhage in 5.7% and symptomatic radionecrosis in 6.2%. Age, RIC grade and ≥ 2 endovascular procedures were risk factors for symptomatic radionecrosis. Due to the unclear benefits of endovascular procedures in addition to GRKS and its potential negative effects, the indication for endovascular treatment should be weighed carefully.

## Introduction

Microsurgical resection is the most validated treatment option for brain arteriovenous malformations (AVMs), especially in a single cure approach on low grade Spetzler-Martin (SM) AVMs. However, a multidisciplinary treatment approach consisting of surgical, endovascular and radiosurgical expertise has been advocated for lesions in eloquent areas or large and complex ones [3, 12]. Gamma Knife Radiosurgery (GKRS) has been shown to be effective in these cases as a single therapy or in a combined approach [8]. Radiation-induced changes (RICs) are the most frequent complications observed after GKRS, which usually occur 1 to 2 years after radiosurgery, as T2 signal changes or perinidal enhancement on neuroimaging follow-up [6]. A grading scale (RIC I-III) has been proposed by Yen et al. to cover the range of severity from T2 signal changes of less than 10 mm to causing mass effect with midline shift [19]. While most RIC regress over time, some may transform into radionecrosis and display cyst formation, chronic encapsulated intracerebral hematoma, and massive edema [17]. Clinical presentation of these changes also varies, ranging from asymptomatic patients to paresis and signs of raised intracranial pressure in need of medical therapy or surgical intervention. Risk of radiosurgical complications is related to the marginal dose value and target volume, and increases as these factors grow. This is more likely to occur in large AVM whose volume exceeds 10 cm^3^, frequently treated with staged-volume strategy [17]. A combination of endovascular treatment and GKRS has been extensively used with the aim of reducing the size of large AVMs prior to GKRS [11]. However, the benefit of this remains controversial as pre-GKRS embolization did not significantly reduce the risk of haemorrhage and permanent neurological deficit [4, 20]. Additionally, the effect of previous endovascular treatments on the incidence of RIC and radionecrosis is unclear. While embolization reduced the risk of symptomatic RICs in one study, it was correlated with cystic formations in another [13, 14].

Giving these uncertainties, the aim of this study is to investigate complications after GKRS for AVMs and predictive factors for symptomatic radionecrosis.

## Methods

A retrospective single center analysis on AVMs treated at our institution by GKRS between April 2008 and December 2016 was performed. Patients with less than 3 years of follow-up or previous treatment at another institution were excluded. AVMs were classified using the Spetzler- Martin (SM) grade and divided in three groups according to their location: lobar, deep and posterior cranial fossa. This study was approved by the local ethics committee and all participants provided written informed consent to scientific research.

All AVMs are discussed interdisciplinary with neuroradiology, neurosurgery and the Gamma Knife team. Typically, inoperable or high-risk AVMs are referred to Gamma Knife. If these AVM’s display high risk features as intranidal aneurysms or high flow parts, targeted embolization is performed prior to Gamma Knife treatment.

### Gamma knife radiosurgery

Radiosurgery was performed using Perfexion® Model (Elekta AB, Stockholm, Sweden) and MRI imaging with T1-weighted contrast-enhanced and T2-weigheted Imaging sequences, as well as DSA using Leksell GammaPlan (Elekta AB, Stockholm, Sweden) were used to delineate the target. A team formed by a neurosurgeon, an interventional neuroradiologist, a radiotherapist and a medical physicist performed treatment planning based on location and size of the AVM. Radiosurgical parameters included target volume, median dose, marginal dose and 12-Gy volume. In patients treated with double session of volume-staged GKRS, values of both sessions were summed up. In patients treated with dose-staged GKRS, only the first session was counted.

### Endovascular treatment

Endovascular treatment was performed with the purpose of targeting intranidal aneurysms and reducing nidus flow, especially for large AVMs, and decreasing the risk of subsequent haemorrhage during the latent period. N-butyl-Cyanoacrilate (NBCA) or non-adhesive copolymer ethylene vinyl alcohol (Onyx; Medtronic, Irvine, California, CA, USA) or both was used. No AVM in this study was treated endovascularly with a curative intention.

### Neuroimaging follow-up and outcome

All patients were clinically evaluated and underwent MRI and MR-angiography (MRA) at 6 months intervals for the first 2 years and annually thereafter. DSA was performed 4 years after GKRS treatment. The absence of nidus filling on DSA was defined as total obliteration of the AVM. In patients who did not undergo DSA at follow up, absence of flow void on MRI or vascular filling on MRA was considered as obliteration.

RICs were evaluated as perinidal hyperintensities on T2-weighted sequence or perinidal enhancement on MRA and graded according to the proposed RIC grading system of Yen et al.: Grade I RICs were mild imaging changes imposing no mass effect on the surrounding brain. Grade II RICs were moderate changes causing effacement of the sulci or compression of the ventricles. Grade III RICs were severe changes causing midline shift of the brain [19]. Symptomatic radionecrosis was defined as radiation induced change persisting over the time of imaging control accompanied by new neurological deficits correlating with imaging. Furthermore, the severity of symptomatic radionecrosis was analyzed and divided into minor (mild to moderate edema with seizures controlled by antiepileptic therapy) and severe (massive edema and cystic formations or encapsulated hematoma with paresis or symptoms of raised intracranial pressure in need of corticosteroid therapy or surgery).

### Statistical analysis

Continuous variables were presented as medians and range (Q1: cumulative percentage of 25%, Q3: cumulative percentage of 75%). Categorical variables were presented as frequency and percentages. The Fisher’s exact test and independent sample median test were used to examine differences between groups. Univariate and multivariate analyses were performed using logistic regression model for prediction of symptomatic radionecrosis. The odds ratio (OR) and 95% confidence interval (CI) were calculated. All analyses were performed using the statistical software program SPSS© version 25.0. Statistical significance was set at *p* < 0.05.

## Results

During the study period 282 patients harbouring 284 AVMs were treated with GKRS. 75 patients were excluded due to incomplete follow up. In total, 209 patients were enrolled in this study. 91 (43%) were SM grade 1–2, 87 (42%) SM grade 3, and 31 (15%) SM grade 4–5. The median neuroimaging follow-up for AVMs treated with GKRS was 54 months. 73 patients (35%) presented with initial bleeding. 63 patients (30.1%) underwent a single endovascular procedure prior to GKRS, while 36 (17.2%) patients were treated with ≥ 2 endovascular procedures. 32 out of 209 patients were treated with double session of GKRS, with either volume-staged (53.2%) or dose-staged (46.8%) techniques. Obliteration of the AVM after GKRS was obtained in 140 AVMs (70%). This was determined by absence of nidus filling on DSA in 113 cases and the disappearance of flow-voids and vascular filling on MRI/MRA in 27 patients. The median time from treatment to obliteration was 48 months (range 18–97 months). Further details are presented in Table 1.

**Table 1:** Patient demographics, arteriovenous malformation (AVM) characteristics, radiosurgical parameters and treatment outcomes

Parameters	Total (*n* = 209)
Median Age	34 (5–76)
Sex	
Male	111 (53%)
Female	98 (47%)
Median FU,month	54 (36–180)
SM grade	
1–2	91 (43%)
3	87 (42%)
4–5	31 (15%)
Lobar	140 (67%)
Posterior Cranial Fossa	21 (10%)
Deep	48 (23%)
Presence of Aneurysm (intranidal/flow-related)	24 (11.4%)
Initial ruptured AVMs	73 (35%)
Single endovascular treatment prior GKRS	63 (30.1%)
≥2 endovascular treatments prior GKRS	36 (17.2%)
Median Marginal Dose, Gy	21 (7–25)
Median Medium Dose, Gy	28.2 (9–56.6)
Median Target Volume, cc	2.9 (0.18–22.58)
Median 12 Gy Volume, cc	7.7 (0.6–50.2)
2 treatments of Gamma Knife radiosurgery	32 (15.3%)
Dose-staged	15 (46.8%)
Volume-staged	17 (53.2%)
Obliteration	140 (70%)
DSA	113 (80,7%)
MRI only	27 (19.3%)
Median duration to obliteration at DSA, months	48 (18.97)

*FU* Follow-up, *SM* grade: Spetzler-Martin grade, *MRI* Magnetic resonance Imaging, *DSA* digital substraction arteriography, *GKRS* Gamma Knife Radiosurgery

### Complications

**Fig. 1:**
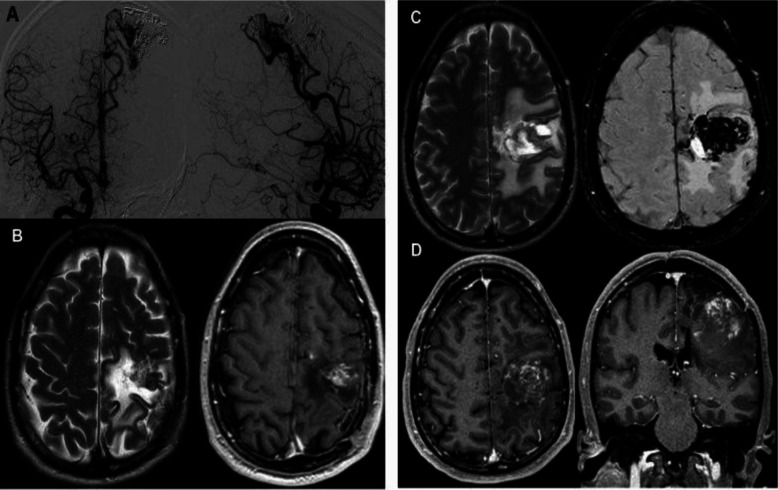
A 50 -year-old men presented with haemorrhage due to a left rolandic-parietal AVM. After 7 endovascular treatments to reduce the size of AVM, the patient underwent GKRS. 4 years post-GKRS the patient complained of chronic headache and seizures. **A**) right internal carotid angiogram showed a residual parietal AVM. **B**) Axial T2 and T1 contrast enhanced 2 years later showed a large heterogeneous well defined radionecrotic tissue with perilesional edema **C**) Axial T2 and SWI images performed during follow-up showed lesion growth with development of cystic formations. **D**) T1 contrast enhanced image in axial view, in the region of the patient's previous left AVM, showed a heterogeneously enhancing lesion, which was surgically removed due to its symptoms and failure of antiedematous therapy

94 patients (45%) developed RICs following GKRS. Among them, 32 (34%) were classified as Grade I, 45 (47.9%) as Grade II, and 17 (18.1%) as Grade III. The median time from GKRS to the development of RICs was 12 months (range 6–45). Symptomatic radionecrosis was found in 13 patients (6.2%). Of these, 8 (3.8%) patients were categorized as minor symptomatic radionecrosis displaying mild to moderate edema as well as epileptic seizures that responded to medical therapy. However, 5 (2.4%) patients developed severe symptomatic radionecrosis with massive edema and cystic formation or encapsulated hematoma. One exemplary case is shown in Fig. 1. Their symptoms ranged from hemiparesis to signs of raised intracranial pressure. Therapy of severe symptomatic radionecrosis was repeated corticosteroid therapy, administration of Bevacizumab as well as surgery in two cases refractory to medical therapy. All cases of severe symptomatic radionecrosis received multiple endovascular treatments prior to GKRS. Furthermore, 12 (5.7%) experienced latent period haemorrhage. An overview of complications after AVM treatment is listed in Table 2.

**Table 2:** Complications after AVM treatment

Parameter	Total (*n* = 209)
Radiation Induced Changes	94 (45%)
Grade I	32 (34%)
Grade II	45 (47.9%)
Grade III	17 (18.1%)
Median duration from treatment to RICs, months	12 (6.45)
Symptomatic radionecrosis	13 (6.2%)
Minor	8 (3.8%)
Severe	5 (2.4%)
Therapy of severe radionecrosis	
Corticosteroids & Bevacizumab	3 (1.4%)
Surgery	2 (1%)
Latent period haemorrhage	12 (5.7%)

### Predictors for symptomatic radionecrosis

In univariate analysis, 12 Gy volume (OR 1.078, CI 1.021–1.138, *p* = 0.007), RIC grade (OR 5.289, CI 2.417–11.572, *p* = < 0.0001) and ≥ 2 endovascular treatments (OR 6.718, CI 2.107–21.421, *p* = 0.001) showed statistical significance in prediction of symptomatic radionecrosis. A multivariate model analysis was performed, including all univariate tested variables. With this approach age (OR 1.058, CI 1.002–1.116, *p* = 0.043), RIC grade (OR 7.323, CI 2.537–21.143, *p* = 0.0002) and ≥ 2 endovascular procedures (OR 14.047, CI 2.585–76.319, p = 0.002) showed statistical significance for prediction. Further details are shown in Table 3.

**Table 3:** Univariate and multivariate ordinal logistic regression analyses for predictors of symptomatic radionecrosis after GKRS

	Univariate analysis		
Factors	OR	CI	*p*
Sex	1.884	(0.595–5.964)	0.281
Age	1.036	(0.998–1.075)	0.064
Target Volume, cc	1.093	(0.976–1.223)	0.123
Marginal dose, Gy	0.982	(0.783–1.232)	0.875
Medium dose, Gy	1.018	(0.947–1.095)	0.623
12 Gy volume, cc	1.078	(1.021–1.138)	**0.007**
Lobar location	2.922	(0.629–13.562)	0.171
Deep location	0.272	(0.034–2.146)	0.217
Initial rupture	0.320	(0.069–1.485)	0.146
SM grade	1.018	(0.455–2.276)	0.965
RICs grade	5.289	(2.417–11.572)	** < 0.0001**
≥ 2 endovascular procedures	6.718	(2.107–21.421)	**0.001**
	Multivariate analysis		
Factors	OR	CI	*p*
Age	1.058	(1.002–1.116)	**0.043**
12 Gy volume, cc	1.060	(0.982–1.145)	0.135
RICs grade	7.323	(2.537–21.143)	**0.0002**
≥ 2 endovascular procedures	14.047	(2.585–76.319)	**0.002**

*SM* grade: Spetzler-Martin grade, *RIC* Radiation induced changes

### Predictors for any RIC or latent period haemorrhage

A multivariate model analysis for any RIC or latent patent period haemorrhage was performed, including all univariate tested variables. For any RIC, female sex (OR 2.128, CI 1.174 − 3.860, *p* = 0.013) and 12 Gy volume (OR 1.081, CI 1.040–1.124, *p* = < 0.001) showed statistical significance for prediction. For latent period haemorrhage, target volume (OR 1.217, CI 1.085–1.365, *p* = < 0.001) was identified as a predictive parameter. Details are shown in Table 4.

**Table 4:** Multivariate ordinal logistic regression analyses for predictors of any RIC and latent period haemorrhage

	Multivariate analysis of any RIC		
Factors	OR	CI	*p*
Sex	2.128	(1.174 − 3.860)	**0.013**
12 Gy volume, cc	1.081	(1.040–1.124)	** < 0.001**
	Multivariate analysis of latent period haemorrhage		
Factors	OR	CI	*p*
Target Volume, cc	1.217	(1.085–1.365)	** < 0.001**

*RIC* Radiation induced changes

###  ≥ 2 endovascular treatment and GRKS as a high-risk group for complications

As ≥ 2 endovascular procedures were shown to be a predictive factor for symptomatic radionecrosis and all severe cases received multiple endovascular treatment, a further analysis of this group was performed. Patients treated with ≥ 2 endovascular procedures + GKRS (*n* = 36) were compared to patients treated with only GKRS or single endovascular procedure + GKRS (*n* = 173). There was no difference in age or gender. Significant differences in terms of SM grade and location were detected.

No significant differences between the presence of initial rupture or aneurysms were detected. In terms of radiosurgical treating parameters, ≥ 2 endovascular procedures + GKRS group showed higher median target volume (5.7 cm^3^, range 0.6–22.58 vs. 2.18 cm^3^, range 0.18–16.2, *p* = < 0.001) and higher median 12 Gy volume (12.25 cm^3^, range 1.8–39.3 vs. 6.4 cm^3^, range 0.6–50.2, *p* = 0,004). No differences in obliteration rates between the two groups were shown. No differences between the incidence of RIC as well as the distribution of RIC grade was found. Symptomatic radionecrosis was significantly more frequent in the ≥ 2 endovascular procedures group (19.4% vs. 3.4%, *p* = 0.02). All cases of severe symptomatic radionecrosis were found in the ≥ 2 endovascular procedures group (13.8% vs. 0%, *p* = < 0.0001). Furthermore, latent period haemorrhage was significantly more frequent in the ≥ 2 endovascular procedures up with 13.8% (*n* = 5) than 4% (*n* = 7) respectively (*p* = 0.037). Further details are listed in Table 5.

**Table 5:** Comparison of baseline demographics data, arteriovenous malformation (AVM) characteristics, radiosurgical parameters, and treatment outcomes between group 1 (≥ 2 endovascular procedures plus GKRS) and group 2 (GKRS only or plus single endovascular treatment)

Factors	≥ 2 endovascular procedures plus GKRS + GKRS (*n* = 36)	GKRS ± single endovascular procedure (*n* = 173)	*p*
Median Age			0.929
Sex			0.583
Male	21 (58.3%)	90 (52%)	
Female	15 (41.7%)	83 (48%)	
SM grade			**0.031**
1–2	13 (36.1%)	81(46.8%)	
3	13 (36.1%)	74 (42.7%)	
4–5	10 (27.8%)	18 (10.5%)	
Location			**0.018**
Lobar	29 (80.5%)	110 (63.5%)	
Posterior Fossa	5 (13.9%)	19 (11.1%)	
Deep	2 (5.6%)	44 (25.4%)	
Initial ruptured AVMs	9 (25%)	64 (37%)	0.185
Intranidal/flow-related aneurysm	1 (2.7%)	23(13.3%)	0.806
Median Target Volume, cm^3^	5.7 (0.6–22.58)	2.18 (0.18–16.2)	** < 0.001**
Median Medium Dose, Gy	27.95 (18.2–54)	28.2 (9.56.6)	0.920
Median Marginal Dose, Gy	20 (15–25)	21(7–25)	0.917
Median 12-Gy Volume, cm^3^	12.25 (1.8–39.3)	6.4 (0.6–50.2)	**0.004**
Obliteration	26(72.2%)	114(65.9%)	0.206
DSA	24(66.7%)	89(78%)	
MRI only	2 (33.3%)	25 (22%)	
RIC	15 (41.6%)	79 (45.6%)	0.715
RIC grade			0.766
1	4(26.7%)	28(35.4%)	
2	7 (46.6%)	38(48.1%)	
3	4(26.7%)	13 (16.5%)	
Symptomatic radionecrosis	7 (19.4%)	6 (3.4%)	**0.02**
Severe	5 (13.8%)	0 (0%)	** < 0.0001**
Latent period haemorrhage	5 (13.8%)	7(4%)	**0.037**

*FU* Follow-up, *SM* grade: Spetzler-Martin grade, *MRI* Magnetic resonance Imaging, *DSA* digital substraction arteriography, *GKRS*: gamma knife radiosurgery, *RIC* Radiation induced changes

## Discussion

Gamma Knife Radiosurgery is an effective tool in the therapy of brain AVMs. Endovascular treatments prior to GKRS are frequently used to reduce flow and volume or to target high risk features. However, the benefit of this remains controversial. On the one hand, endovascular treatments before GKRS reduces the volume of an AVM, allowing to use a higher irradiation dose to the margin of a smaller target volume with better obliteration rate and fewer complications [1]. On the other hand, recent studies stated disadvantages of endovascular treatments. Embolic material reduces the AVM nidus delineation and hypoxia leads to reduced radiosensitivity in the AVM tissue while angiogenic activity increases [5, 9]. Moreover, the effect of prior endovascular treatments on GKRS complications such as radiation induced changes/radionecrosis is unclear. Therefore, we analysed in this present study a single centre experience on complications after GKRS for AVMs and possible predictive factors for symptomatic radionecrosis.

Radiation induced changes are a frequent complication after GRKS reported with an incidence of 16–62% [19]. In this present study, we documented 45% RICs with 47.9% grade II and 18.1% grade III changes. While most RIC were reversible, 13 patients (6.2.%) showed symptomatic radionecrosis during their follow-up. This is in accordance with data found by Pollock et at., where they reported a rate of 6.9% [15]. Predictive factors for the appearance of RICs were reported and included marginal dose, target volume, eloquent location, AVM angioarchitecture, history of rupture, obliteration rate and embolization [2]. For long term complications, predictive factors were described as early RIC, AVM obliteration, higher maximal GKRS dose, large nidus volume, lobar location as well as longer follow-up [7, 15, 16]. Pan et al. examined 20 cases of cystic formations in their study group of 1203 AVMs. This study analysis found that prior endovascular treatments and RICs grade were correlated with cystic formations [14]. This appears consistent to the results of our univariate and multivariate analysis, where risk factors for symptomatic radionecrosis were age, higher RIC grade and ≥ 2 endovascular procedures.

As all severe cases of symptomatic radionecrosis received multiple endovascular treatments and ≥ 2 endovascular procedures were shown to be a predictive factor, we further analysed a high-risk group for complications consisting of patient with ≥ 2 endovascular procedures. These AVMs were more frequently of higher SM grade and interestingly less frequently located deep. Also, higher target and 12 Gy volume was used. Interestingly, no difference in obliteration rate and RIC was found. Symptomatic radionecrosis was more frequent in this group. Furthermore, this group was also at higher risk for latent period haemorrhage.

The pathogenesis of RIC and radionecrosis unclear. Pathological examination of these lesions has revealed: edema, reactive gliosis, blood vessel dilation, endothelial thickening, and disruption of capillary structures [10]. In turn, the damage to capillary walls determines protein exudate, fibrinoid necrosis and microhaemorrhage [16, 18]. Therefore, prior AVM haemorrhage and endovascular treatment may facilitate cyst formation increasing tissue vulnerability and creating a hypoxic environment, leading to the development of fragile vessels [13, 14]. These could explain the correlation between symptomatic radionecrosis and multiple endovascular treatments prior GKRS reported in this study.

However, there is insufficient data to conclude that the higher rate of complication is a consequence of multiple endovascular treatments as it may be a consequence of AVM morphology.

Furthermore, it is important to note that even though all patients were treated with the same endovascular treatment strategy, the data on endovascular treatment (e.g. type of embolic agent, number of feeders) was too heterogenous to be processed in a statistical useful manner. Therefore, we cannot exclude correlations between specific endovascular treatment parameters and complications.

Nevertheless, in addition to the unclear benefits of endovascular treatments before GKRS in the literature, this uncertainty led to a strategy change at our institution towards a more cautious approach regarding multiple upfront endovascular treatments, especially in incidentally found asymptomatic AVMs in young patients, to limit the future impact of radiation throughout the lifespan.

## Conclusion

Complication after GKRS for AVMs were latent period haemorrhage in 5.7% and symptomatic radionecrosis in 6.2%. In our series, age, RIC grade and ≥ 2 endovascular procedures increased the risk for symptomatic radionecrosis in multivariate analysis. Due to the unclear benefits of endovascular procedures in addition to GRKS and its potential negative effects, the indication for endovascular treatment should be weighed carefully.

## Study limitations

This study has several limitations. First, it is a retrospective single centre study. Second, all patients were treated with the same endovascular treatment strategy. However, while therapy strategies are in general uniform at our institution, heterogenous decisions concerning endovascular treatments cannot be excluded. Furthermore, detailed data on endovascular treatment was too heterogenous to be processed in a statistical useful manner. Therefore, we cannot exclude correlations between specific endovascular treatment parameters and complications. Third, symptomatic radionecrosis is rare and therefore low patient numbers can reduce the value of such a study.

## Data Availability

No datasets were generated or analysed during the current study.
